# Bacteriophage biodistribution and infectivity from honeybee to bee larvae using a T7 phage model

**DOI:** 10.1038/s41598-018-36432-x

**Published:** 2019-01-24

**Authors:** Henrique G. Ribeiro, Rossana Correia, Tiago Moreira, Diana Vilas Boas, Joana Azeredo, Ana Oliveira

**Affiliations:** 1 CEB - Centre of Biological Engineering, LIBRO - Laboratório de Investigação em Biofilmes Rosário Oliveira, University of Minho, 4710-057 Braga, Portugal; 2 I3S - Institute for Research and Innovation in Health Sciences, University of Porto, 4200-135 Porto, Portugal; 3 Ipatimup - Institute of Molecular Pathology and Immunology of the University of Porto, 4200-135 Porto, Portugal; 4 BeePrado Unipessoal, Lda., Rua 1, no 32, Ramalha, 4730-475 Vila de Prado, Portugal

## Abstract

Bacteriophages (phages) or viruses that specifically infect bacteria have widely been studied as biocontrol agents against animal and plant bacterial diseases. They offer many advantages compared to antibiotics. The American Foulbrood (AFB) is a bacterial disease affecting honeybee larvae caused by *Paenibacillus larvae*. Phages can be very significant in fighting it mostly due to European restrictions to the use of antibiotics in beekeeping. New phages able to control *P. larvae* in hives have already been reported with satisfactory results. However, the efficacy and feasibility of administering phages indirectly to larvae through their adult workers only by providing phages in bees’ feeders has never been evaluated. This strategy is considered herein the most feasible as far as hive management is concerned. This *in vivo* study investigated the ability of a phage to reach larvae in an infective state after oral administration to honeybees. The screening (by direct PFU count) and quantification (by quantitative PCR) of the phage in bee organs and in larvae after ingestion allowed us to conclude that despite 10^4^ phages reaching larvae only an average of 32 were available to control the spread of the disease. The fast inactivation of many phages in royal jelly could compromise this therapeutic approach. The protection of phages from hive-derived conditions should be thus considered in further developments for AFB treatment.

## Introduction

Bacteriophages (phages) are viruses that exclusively infect bacteria and are highly specific for their hosts. They take advantage of bacteria biosynthetic machinery by directing it toward the synthesis of more phages able to induce the bacterial lysis and phage release to the environment. New infection cycles will be triggered as soon as phages successfully reach available hosts nearby. The binding of phage virions to specific bacterial receptors with subsequent bacterial death makes them highly specific antibacterial agents^1^. Their self-replicating properties enable that efficient concentrations of phage particles are achieved at the site of infection at curative doses^2^. Phages are also recognised by the inability to infect eukaryotic cells showing no toxicity to plants, animals or humans^3^. It is estimated that 10^31^ phages occur in the biosphere^4^.

All these described features make phages a promising strategy to control bacterial infections, including American Foulbrood (AFB). AFB is one of the most devastating bacterial diseases affecting honeybee larvae worldwide^5^. This contagious disease is caused by the vegetative form of the Gram-positive bacterium *Paenibacillus larvae* and is easily spread trough their highly resilient spores. The sporulated form of the bacteria withstand extreme temperatures and exposure to disinfectants, tolerate antibiotics and is able to remain dormant for years^6^.

The treatment of AFB represents an important challenge to the pharmaceutical industry as the hive-derived products (honey, propolis, royal jelly, bee venom and bee pollen) cannot be commercialised if contaminated with antibiotics (Regulation (EEC) 2377/90 and further amendments)^7^. Furthermore, bacterial resistance to the commonly prescribed antibiotics^8,9^ contributes to reduce the available alternatives to the conventional burning of hives for *P. larvae* control.

AFB infection process begins when adult bees provide spore-contaminated food to their larvae that become infected during the first instars (around 48 hours after the egg hatching)^10^. As soon as spores arrive to larvae midgut they germinate into vegetative cells and massively proliferate^11,12^. In the last stage of the infection *P. larvae* breach the midgut epithelium and invade the larval haemocoel resulting in larval death^13^. After depletion of the nutrients *P. larvae* starts sporulation and billions of spores are released inside the hives that easily spread across apiaries^14^.

Efforts have been made to explore the potential of phage therapy for treating AFB by evaluating phage action against *P. larvae*^15–19^. Recently published studies report the use of spore-infected laboratory-raised larvae to evaluate phage effectiveness in decreasing larvae mortality. In those studies larvae were fed directly with phages eventually simulating the spray administration to larvae combs. The success of a prophylactic treatment of larvae with phages before spore infection was assessed^16,18^, but no consensual results were obtained concerning the efficacy of treating larvae after infection^16–18^. More recently, in tests performed in experimental hives, Brady *et al*.^19^ reported that larvae sprayed with phages were effectively protected and rescued from *P. larvae* infections. The phage delivery in the adult bee food might though be a more feasible strategy toward hive management causing lower phage waste. Hence, a thorough assessment of phage biodistribution and bioavailability from adult bee to young larvae was performed in this work relying on the bee social organization for brood rearing.

## Results

### Preliminary *in vitro* analysis

Before undertaking the *in vivo* assays the T7 phage stability in a 50% (w/v) sucrose solution (routinely used for feeding bees) was assessed *in vitro* for 24 hours. The results revealed that, at least in this time period, the phage viability was not affected (see Supplementary Fig. S1). In order to prevent phage overestimation in further analysis, the screening for phages infecting *E. coli* BL21 (the bacterial T7 host that if present is able to amplify the phage) and for other T7 phage host strains was undertaken in samples obtained from experimental colonies. Both types of analysis revealed the absence of other hosts or phages in the hive (data not shown).

### Biodistribution assay

*In vivo* assays were conducted in 6 different bee colonies. Adult bees were fed with a T7 phage suspension prepared in 50% (w/v) sucrose. After 24 hours, larvae were grafted -  together with the surrounding royal jelly (RJ) that was removed from larvae by washing - and stored. Adult bees were also collected, separated into abdomens (Abd), heads (H) and thoraxes (T) and dissected. Heads and thoraxes (H&T) were then mixed as they hold together the first part of the foregut. Before that, in order to target only phages present inside their organisms, both adult bees and larvae were washed with saline solution (0.9% (w/v) NaCl). No phage viability loss related with the sample homogenisation with glass beads was observed.

**Figure 1:**
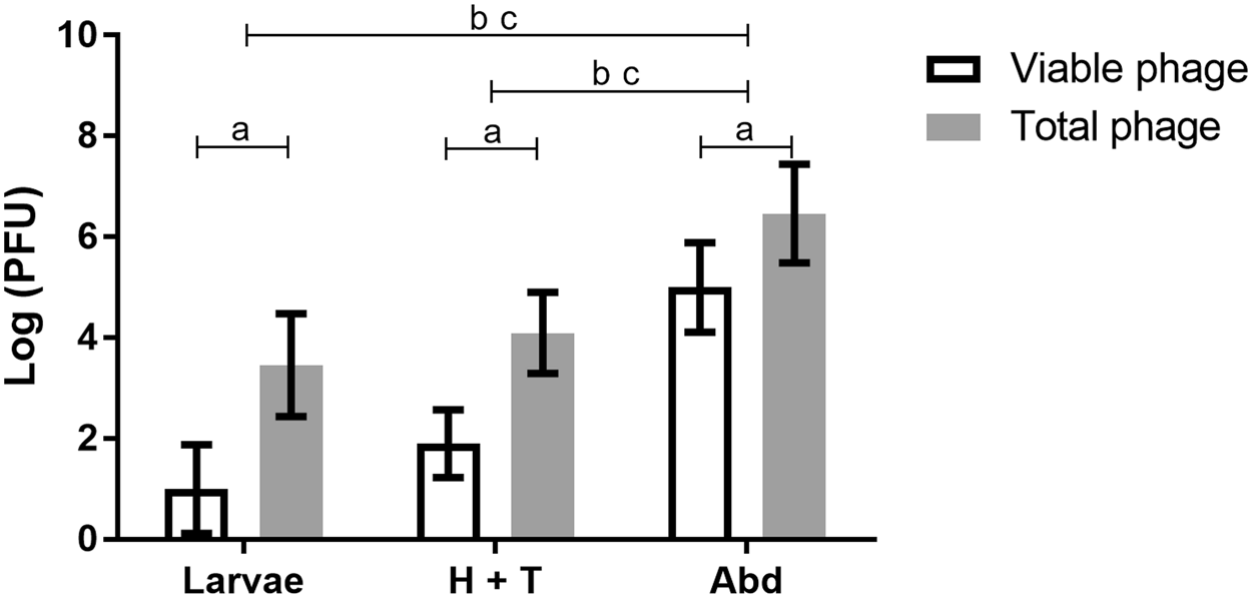
T7 phage (PFU) per section of adult bee (H&T and Abd) and per bee larva, 24 hours after providing phage (10^9^ PFU.mL^−1^ in 50% (w/v) sucrose) in bee’s feeders. Each column represents the mean of six independent *in vivo* experiments (n = 6) and error bars indicate the standard deviation (SD). Statistical significance (p < 0.05) is indicated above the columns by “a”, “b” or “c”: a - differences between viable and total phage; b - differences considering viable phage. c - differences considering total phage.

The biodistribution of T7 in adult bees and in bee larvae was assessed by enumeration of the plaque forming units (PFU) in *E. coli* BL21 lawns, which indicates viable phages present inside the organism (“viable phage”) and by quantitative PCR (qPCR), which gives a quantitative estimation of the total amount of phages (“total phage”) (Fig. 1). Results obtained by PFU count revealed that the average amount of viable phages detected per Abd of adult bee (2.8 × 10^5^ PFU/bee) was significantly higher (p < 0.05) than per H&T (175 PFU/bee). The average quantity of phages per larvae was 32 PFU/larvae, lower than the observed in H&T, but this difference was not statistically significant (p > 0.05).

The amount of phages per bee section or per larva detected by qPCR (calculated by equation (2) of section 4.4.4) was higher than by direct PFU enumeration (p < 0.05) (Fig. 1). Both methods revealed the same average trend between samples: the amount of phages recorded in the Abd (1.2 × 10^7^ phage/bee) was higher than in the H&T (4.2 × 10^4^ phage/bee) and higher than in the larvae (1.4 × 10^4^ phage/larvae). The two latter groups revealed again no meaningful differences between them (p > 0.05).

In each qPCR reaction an internal amplification control (IAC) was used to avoid false negative results and to assess phage DNA purification efficiency. It should be mentioned that the rate of IAC DNA recovery after sample treatment was lower in H&T and Abd. Therefore records for total phage recovery might be less accurate in adult bee than in larvae.

**Figure 2:**
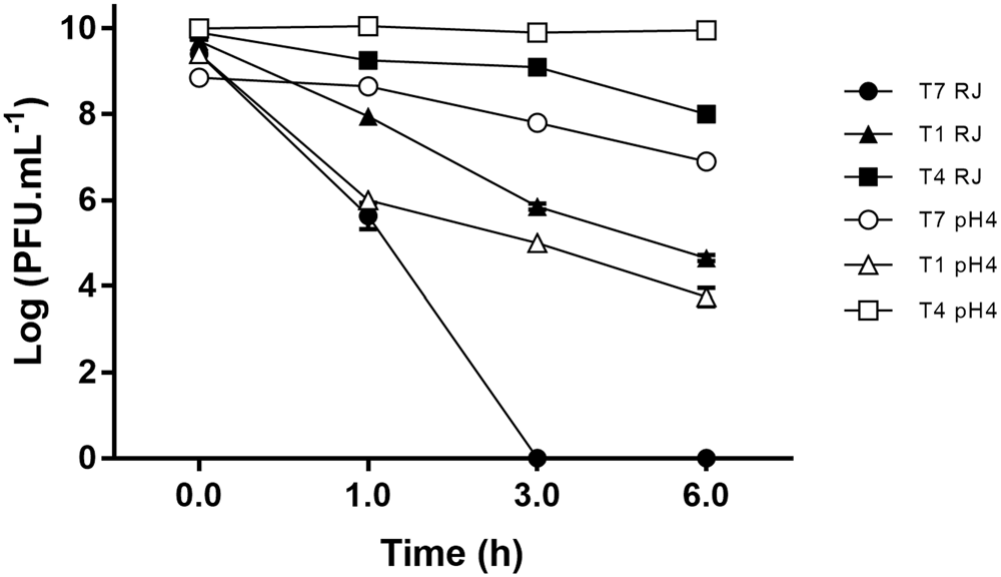
Effect of commercial Royal Jelly (pH 4.0) on the viability of T7 (dark circle), T1 (dark triangle) and T4 (dark square) phages (PFU.mL^−1^). The control in Universal buffer at pH 4.0 is also plotted for each phage (correspondent white figures). Limit of Detection = 2 Log; statistical significance, p < 0.05.

When surrounding larvae RJ was analysed no viable phages were present. Nevertheless, in average, 8.3 × 10^6^ phage.mL^−1^ were detected by qPCR. The effect of RJ in phage viability was assessed through the incubation of phage in a commercial RJ. Phage particles did not persist infective for more than 3 hours (Fig. 2). The assumption that this inactivation could probably be due to the low pH of RJ led us to the monitoring of the phage concentration using an Universal buffer solution at pH 4.0 (the same pH of RJ). T7 phage showed a higher tolerance to the buffered solution comparatively to RJ dropping only about 2-Log PFU.mL^−1^ in 6 hours. The same *in vitro* assay was performed for two other well-described phages from different taxonomic genus T1 and T4, in order to support the interpretation of T7 phage data (Fig. 2). Both phages revealed to be more stable in RJ than T7 phage, however, the latter preserved its viability for longer comparatively to T1 phage in the buffered solution at pH 4.0. After 6 hours in RJ, T1 and T4 phage decreased about 5 and 2-Log PFU.mL^−1^ respectively (p < 0.05). Besides pH 4.0, the stability of these phages under pH 3.5 and 4.5 (also reported for RJ)^20^ was assessed. Similar results were obtained: T4 was the most stable phage followed by T7 and then by T1 phage (see Supplementary Fig. S2).

### Phage staining in larvae tissue

To confirm phage presence in the larvae tissue, an immunofluorescence assay was conducted. Microscopic observations were performed after incubating samples with a T7 phage antibody conjugated with Alexa Fluor® 488.

**Figure 3:**
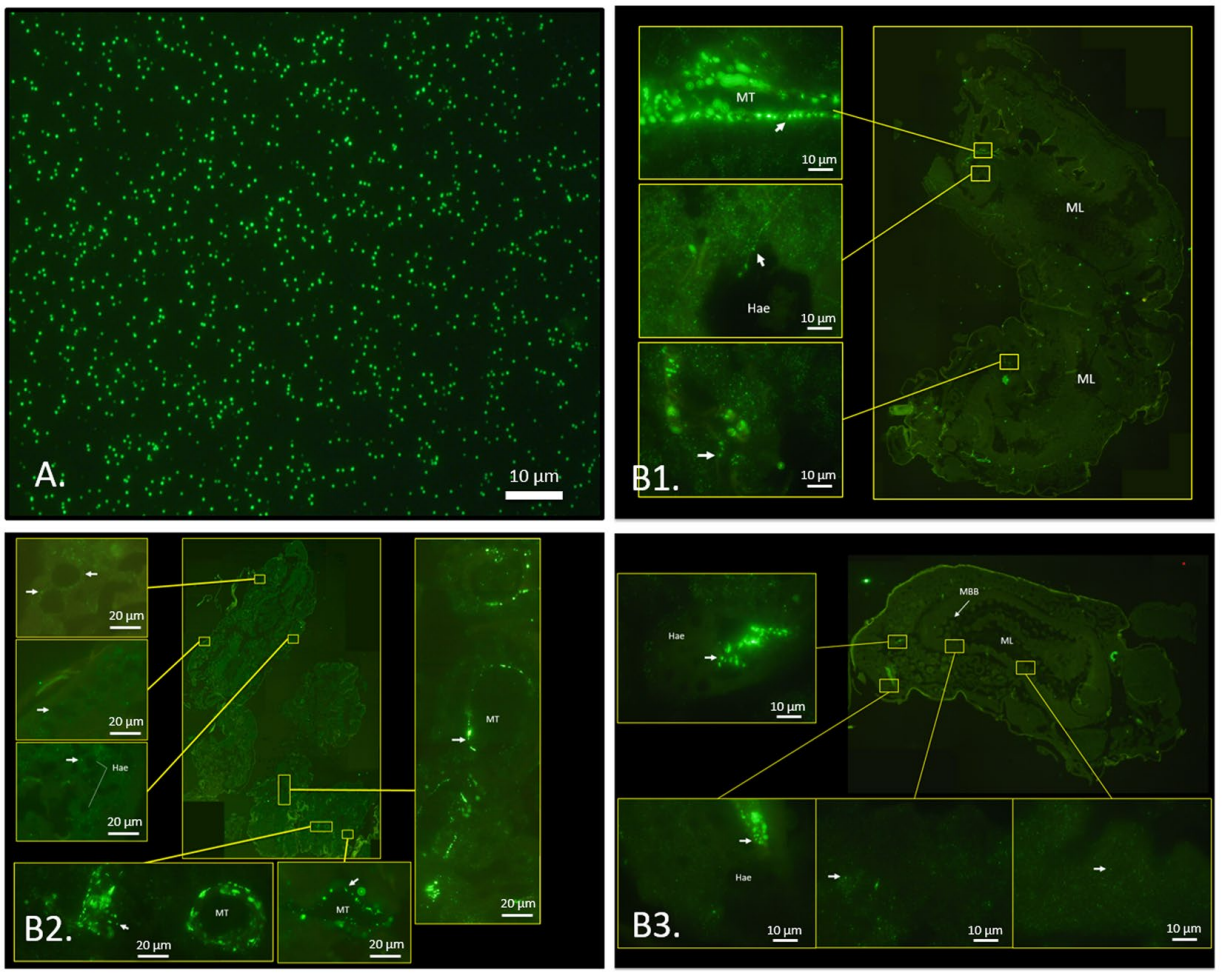
Immunofluorescence staining with T7 tag polyclonal antibody and Goat anti-Rabbit IgG (H + L) Cross-Adsorbed Secondary Antibody conjugated with Alexa Fluor® 488. (**A**) Positive control composed by a 10^8^ PFU.mL^−1^ T7 phage suspension. (**B**) Larvae sections (1^st^ to 3^rd^ instars) for localization of T7 phage (B1: cross section; B2 and B3: longitudinal sections). Each picture shows a global image and emphasizes some details marked with a yellow square. ML: midgut lumen; MBB: midgut brush border; MT: Malpighian tubes; Hae: Haemocoel. Phages are shown as bright green pixels (white arrows) alone or aggregated, depending on the displayed brightness of the dots. Photomicrographs were obtained with 1000× total magnification.

Figure 3A shows a stained 10^8^ PFU.mL^−1^ T7 phage suspension and allowed to identify strong bright green pixels randomly distributed in the microscopic field (FITC filter). The same bright green signal was observed in tissues from larvae collected 24 hours after they were fed to adult bees (indicated by white arrows in Fig. 3B). This signal was found in tissues around haemocoel, cavities in between organs whereby the hemolymph circulates (identified in the images as dark circular empty spaces between tissues), on the midgut brush border and with higher intensity in the epithelium of Malpighian tubes (responsible for osmotic and ionic regulation in larvae). The TRITC filter was used in all observations to distinguish the T7 phage-specific bright-green fluorescent signal of Alexa Fluor® 488 from the tissue bright-green autofluorescence.

Some of the details highlighted in the Fig. 3 are present in Supplementary Fig. S4 and show that bright green pixels observed in the images obtained with the FITC filter are not present in images from the same microscopic field, captured with the TRITC filter. This is evident in the overlay of FITC and TRITC images, demonstrating that the green dots of the overlapped images are exclusively phages.

Additional observations were performed on samples from the larvae that did not receive phages (negative control). In this case, the image captured by FITC presented the same level of brightness when compared with TRITC filter, revealing the tissue autofluorescence and absence of T7 phage-specific bright-green fluorescent signal of Alexa Fluor® 488. When combining images obtained with both filters no bright-green fluorescent signal was observed (see Supplementary Fig. S3).

## Discussion

The work presented herein plans to clarify phage biodistribution and bioavailability from adult bee to young larvae, particularly when phages are administered orally to adult bees. The purpose was to understand the potential of this delivery strategy to effectively provide viable phage to larvae.

Despite recognizing that spraying hives might be effective in the pathogen control, authors consider that the use of phage suspended in adult bees’ food may be more advantageous. It seems easier to include it in hive management, it’s less time consuming and causes lower product waste.

### Phage biodistribution and stability in adult bees

The phage distribution analysis in adult bees revealed that the amount of phages detected in bee Abd was higher (p < 0.05) than the obtained in H&T. These differences may be explained not only by the size of gut compartments present in each bee section but also by the influence of peristalsis that rapidly moves the food down the gut.

Phage inactivation or phage efficacy impairment occurring inside the honeybee might be due to hostile conditions that phages have to face after being ingested. In fact, the insect gut poses multiple challenges for microorganisms and viruses ingested with the food. That might include unfavourable pH or harmful digestive enzymes^21^.

T7 phage is known to be stable between pH 6 and 8^22^. Most of the bee compartments from the Abd (mid and hind gut) are not acidic - the pHs range from 7 to 7.4. The crop (foregut) though stores substances such as nectar and honey whose pH ranges between 5.6 and 6.1^23^. This acidic effect of the crop may indirectly influence phage stability also in H&T due to trophallaxis. In this action the crop content is regurgitated back to the mouth^24^. We hypothesize that not only pH but also enzymes with proteolytic activity present in bee gut may contribute to phage titre loss. Peptidases such as trypsine and chymotrypsine are present in the midgut where pollen is thought to be broken down. None of these enzymes are found in the crop. This indicates that the proteolysis bias on phages may only occur in Abd^25,26^.

Phage impairment in H&T might also be due to the presence of RJ, whose antiviral activity had already been reported^7,27^. This substance is produced in the head by the hypopharyngeal glands. The regurgitated crop content is mixed with hypopharyngeal glands’ secretions in the first part of the digestive tube, before being deposited in larvae combs. When surrounding larvae RJ was analysed, phages though present lost their infectivity. This drop of viability was confirmed *in vitro*. The T7 phage did not remain infective for more than 3 hours after immersed in commercial RJ (Fig. 2).

Gochnauer^28^ had already reported phage sensitivity to hive conditions. According to this research phages could not survive if stored in larval food for long periods of time. They needed to be ingested rapidly by larvae in order to reach the nearly neutral alimentary environment (pH 6.8) in which phage might have been stable^29^. Yost *et al*.^18^ also describes inability to recover viable phages from larvae that were artificially fed with RJ mixed only with phage (without host for replication). The negative results reported by Yost *et al*. (2016) could be due to phage inactivation by RJ before reaching larvae.

The fact that all phages tested were more tolerant to pH 4.0 than to RJ suggests that the low pH occurring in RJ (3.4–4.5)^20^ is not the only factor affecting phage stability^22^. The presence of substances such as phenolic compounds^7^ and proteinase(s)^30^ might contribute to phage inactivation probably through interaction with phage structural proteins^31^.

The type of phage used in the therapy may also influence its behaviour in general hive-derived conditions: T1 and T4 phage survived for longer periods than T7 in RJ (Fig. 2). This tendency doesn’t seem to be only related to pH sensitivity because T1 was the most susceptible phage at pH 4.0 (see Supplementary Fig. S2).

### Phage biodistribution in larvae

Biodistribution assays revealed that phages remain active in adult bee tissues and immunohistochemical analysis confirmed phage transfer from bee to larvae. Microscopic images revealed a wide phage distribution in larvae tissues (Fig. 3A). After being uptaken by larvae, phages not only reached the midgut lumen but they also penetrated the epithelium. They might have spread then to the haemolymph, as they were found in tissues around the haemocoel. This was confirmed by their presence in Malpighian tubes, which are structures of the excretory system.

However, as discussed above, RJ contains factors that inactivate phages within a 3 hours period. The exposition of phage particles to RJ before being uptaken by larvae might have led to a massive phage inactivation. This accounts for the very low amount of active phage particles present in larvae guts.

Larval midgut conditions (pH around 7^14^ and absence of proteolytic enzymes^25^) are apparently harmless to phages.

Previous reports state that the uptake of around 10 spores are enough to successfully initiate infection in the larvae^32,33^. Nevertheless, this dosage-mortality relationship seems to depend on larval age, genetic constitution and bacterial strain^12,34^ and may vary with a factor of at least 20^34^. Based on previous reports of exposure bioassays in artificially reared honey bee larvae^12^, 100 to 800 colony forming units (CFU) per mL *P. larvae* cells - about 4 to 40 CFU per larvae - may initiate an infection that will kill 50% of the larvae tested (LC_50_) depending on the virulence of the strain. According to the reported infectious doses, the 32 active phage particles wouldn’t most likely be sufficient to effectively control the disease.

In conclusion, this work provides evidence that phages administered in bee food are successfully uptaken by the bee and transported in their organs, reaching larvae through the bee-larvae feeding chain. Nevertheless, the amount of viable particles found in larvae seems to be very low to be able to reduce *P. larvae* load and thus to control AFB.

The improvement of the oral delivery effectiveness in the AFB therapeutic might be achieved not only by screening for phages with high tolerance to RJ, but also by the development of strategies that protect phage from general hive-derived conditions or by engineering phages to endure this harsh environment.

## Material and Methods

### Phage production

*E. coli* BL21 (Stratagene) was the strain used as T7, T1 and T4 phage propagation strain. T7 phage was gently provided by J. Molineux (University of Texas), and T1 and T4 phages by Stan Brouns (Delft University of Technology). Bacteria were cultured at 37 °C overnight (O/N) in Tryptic Soy Broth (TSB, VWR) or Tryptic Soy Agar medium (TSA; TSB containing 1.5% (w/v) agar, NZYTech). For phage propagation, 5 μL of phage suspension were spread evenly on host bacterial lawns using a paper strip and incubated O/N at 37 °C. Then, 3 mL of SM Buffer (5.8 g.L^−1^ NaCl, 2 g.L^−1^ MgSO_4_.7H_2_O, 50 mL.L^−1^ 1 M Tris-HCl pH 7.5, VWR) were added to each plate and re-incubated O/N at 4 °C with gentle stirring (50 rpm on a PSU-10i Orbital Shaker (BIOSAN)). The floating liquid was collected, centrifuged (10 min, 9,000 × g, 4 °C), and purified with 1:4 (v/v) chloroform followed by filter sterilization (PES, GE Healthcare, 0.2 μm). Phage suspension were stored at 4 °C until use.

### Phage viability in sucrose and royal jelly

Before *in vivo* experiments, envisaging honeybees feeding, the effect of 50% (w/v) sucrose solution in T7 phage viability was evaluated. After field trials, in order to support *in vivo* results, the influence of a commercially available RJ (pH 4.0) (Apiguarda, Portugal) in T7 phage infectivity was monitored with time. This was done also for T1 and T4 phages to support data obtained for T7 phage. Universal Buffer solutions (150 mM KCl, 10 mM KH_2_PO_4_, 10 mM Na-Citrate and 10 mM H_3_BO_3_) adjusted with HCl to have pH of 3.5, 4.0 and 4.5 were prepared in order to assess phage behaviour in the pH ranges reported for RJ, including the used herein. The final phage concentration was 10^9^ PFU.mL^−1^. The incubation in sucrose was performed at room temperature (RT) and the incubation in RJ and buffered solutions at 37 °C with 5% CO_2_, to mimic hive conditions. Samples were collected at 0, 6, 9, 12 and 24 hours in the first case and at 0, 1, 3 and 6 hours in the others. In each time point, three independent samples were taken and the titration was performed as described above.

### Screening for T7 phage and *E. coli* host strains in experimental hives

In order to assure that the phages present in hives were only provided by artificial feeding the screening for other phages able to infect the *E. coli* strain used in this study was performed. The presence of other bacterial strains that could be sensitive to T7 phage was also examined in the colonies. These procedures intended to avoid phage overestimation. For that, samples were collected from empty and brood combs of each colony, using swabs soaked in 0.9% (w/v) NaCl. For phage detection, swabs were immersed in an *E. coli* Bl21 early-grown suspension (about 6 hours), incubated O/N at 37 °C (120 rpm) and after that, the suspension was filter-sterilised (0.22 µm). A drop of 10 µL of the filtrate was placed onto 0.6% TSA agar previously inoculated with 100 µL of an *E. coli* BL21 suspension, and re-incubated at 37 °C, O/N. For *E. coli* detection, swabs were streaked in TSA and MacConkey agar (Merck-chemicals), followed by an O/N incubation at 37 °C. Plates were searched for colonies and in the case of positive results, colonies were suspended in TSB and tested for phage sensitivity as described.

### Biodistribution assay

#### Experimental model

The experimental apiary used for this *in vivo* experiment was located in the north of Portugal (Vila Nova de Famalicão) where six different colonies of *Apis mellifera iberiensis* with the same dimension and under the same development state were housed.

For phage administration, 1 × 10^9^ PFU.mL^−1^ suspended in a 50% (w/v) sucrose solution were provided to bees’ feeders. After 24 hours, 30 adult bees and 15 larvae from 1^st^–3^rd^ instar were collected from each colony (larvae were carefully grafted into a microtube together with the surrounding RJ with the aid of a larvae-picking tool).

Both adult bees and larvae were carefully washed 3× with saline solution (0.9% (w/v) NaCl) before processing. Bees were treated as follows: after removing their wings and legs they were divided in three parts: head, thorax and abdomen. The content of each part was carefully removed with the aid of two sterile forceps: heads and thoraxes’ content (H&T) were mixed together; guts were removed form the abdomens (Abd) and treated separately. Larvae embedded in RJ were weighted (L_RJ). After the first washing the decanted supernatant (which volume was recorded as FW) was recovered for phage titration and larvae weight (L) was recorded. The dilution rate (w/v) of RJ (obtained from L_RJ (g) – L (g)) in FW was assessed for further calculations.

All the samples, except RJ, were well homogenised in 0.9% (w/v) NaCl (the volume used was recorded for further calculations) with glass beads, by vortexing. Samples were stored at 4 °C for no more than 30 minutes for further analysis. In a previous assay, T7 phage suspended in SM buffer was similarly homogenised to exclude the possibility of phage viability loss due to the sample processing.

#### Determination of viable T7 phage in bees and larvae

Phage counts were performed in 50 µL of bee (H&T and Abd) and larvae homogenates. Each sample was mixed with chloroform (5:1 (v/v)) homogenised by vortexing and centrifuged at 8000 × *g*, 4 °C, for 3 minutes. The upper phase was collected carefully to a new tube and phage titration was done based on the double agar overlay technique^35^, as previously described. This analysis was also performed in the first larvae washing (diluted RJ).

#### DNA extraction and purification from biological samples

T7 phage DNA present in the above treated samples was assessed by qPCR in a CFX96 thermal cycler (Bio-Rad) and for that two different genes with published sequences were targeted in separate reactions: the T7 major capsid protein (MCP) gene for phage detection and the *E. coli LacZ* gene for the internal amplification control (IAC), used to avoid false negative results and to assess phage DNA purification efficiency. Primers (Table 1) were designed using SnapGene™ software (version 1.1.3) (www.snapgene.com).

**Table 1:** Primer sequences used in qPCR, amplicons’ size of the PCR products and amplification efficiency of qPCR reactions.

Target gene	Sequence (5′-3′)	Amplicon size (bp)	Amplification Efficiency (%)
T7 MCP	F: CCGCAACGTTATGGGCTTTG R: CTCACCTTTATTGGCAGGGAAG	119	93.6
LacZ (IAC)	F: AGCGAAACCGCCAAGACTGTTA R: GTGGATGAAGACCAGCCCTTCC	135	83.6

The DNA was purified from the previously homogenised samples of H&T, Abd, larvae and from diluted RJ. Before DNA extraction, samples were supplemented with 25 ng.µL^−1^ of the IAC template (a 3075 bp purified amplicon originary from *E. coli LacZ* gene). Zymo Quick-DNA™ Viral Kit was used for DNA purification, with some modifications relatively to the manufacturer instructions: a 3 hours period of incubation with the supplied Lysis Buffer and the elution in 15 µL of the supplied Storage Buffer.

#### T7 phage quantification in bees and larva

In order to quantify the IAC concentration present in the purified DNA, a standard curve was obtained (Cq_IAC_ = −3.781 × Log [IAC] (ng.µL^−1^) + 8.369 (Cq = quantification cycle)) using several concentrations of the *LacZ* amplicon (5.5, 0.55, 0.11, 0.055 ng.µL^−1^). For that 5 µL of SsoFast™ EvaGreen (BioRad), 2 µL of DNA template, 1 µL of a 5 µM LacZ (IAC) primer mix and HyPure^TM^ Molecular Biology Grade water (GE, Healthcare) up to 10 µL were mixed and run for 3 minutes at 95 °C followed by 40 cycles of 10 seconds at 95 °C, 10 seconds at 58 °C, and 10 seconds at 65 °C (the melt curve was generated by heating from 65 to 95 °C with increments of 1 °C, 5 seconds dwell time).

The assessment of the DNA extraction efficacy was obtained through equation (1) that was used to normalise T7 phage concentration obtained from biological samples.

1 $$100-((25\,{\rm{ng}}.\mu {{\rm{L}}}^{-1}{[{\rm{IAC}}]}_{{\rm{sample}}}\,{\rm{ng}}.\mu {{\rm{L}}}^{-1})/25\,{\rm{ng}}.\mu {{\rm{L}}}^{-1})\ast 100$$

To assess the amplification efficiency of the primer pair T7 MCP another standard curve was defined: Cq_MCP_ = −3.486 × Log [T7 MCP] (ng.µL^−1^)+11.467). T7 phage commercial DNA (BIORON) serial dilutions (0.3; 0.1; 0.03; 0.01 ng.µL^−1^) were used as template for the qPCR reaction mixture. The annealing temperature, in this case was 54 °C.

In order to estimate T7 phage concentration (PFU.mL^−1^) in the treated samples, the DNA of five serial dilutions of a 10^8^ PFU.mL^−1^ T7-phage suspension was purified (Zymo Quick-DNA™ Viral Kit), and used as template for qPCR analysis (T7 MCP primers). The standard curve equation was obtained (2). A negative control using HyPure^TM^ water was included in each reaction and three replicates of each condition were analysed in all the assays.

2 $${{\rm{C}}{\rm{q}}}_{{\rm{M}}{\rm{C}}{\rm{P}}}=-3.3686\times \,{\rm{L}}{\rm{o}}{\rm{g}}\,[{\rm{T}}7\,{\rm{p}}{\rm{h}}{\rm{a}}{\rm{g}}{\rm{e}}]\,({\rm{P}}{\rm{F}}{\rm{U}}.{{\rm{m}}{\rm{L}}}^{-1})+43.06$$

#### Immunohistochemical targeting of T7 phage in larvae bees

For preparing samples for sectioning 10 larvae were collected from the experimental hive before the beginning of the *in vivo* experiment and used as negative controls. The treated group was composed on 30 larvae picked 24 hours after phage administration. For tissue fixation, all the larvae were previously perfused: 10% (v/v) buffered formalin was introduced with the aid of a perfusion needle. After 24 hours, samples were routinely processed in an automated system and embedded in paraffin. Then, sequential sections of 4 μm were made in a paraffin microtome (Microm HM335E) and placed in adhesive microscope slides (Superfrost®, Sigma).

Before immunohistochemical procedures, sections were dewaxed through immersion in xylene (Fisher Chemical, Loughborough, UK) and sequentially re-hydrated in 100%, 80%, and 50% (v/v) ethanol (Panreac). After rinsed with distilled water slides were allowed to air dry.

Prior to incubation with specific antibodies samples were permeabilised with 0.1% Triton™ X-100 (Sigma) in Phosphate Buffered Saline 1×(PBS, Sigma) for about 10 minutes. Tissue samples were blocked with 5% (w/v) bovine serum albumin (BSA, Sigma) in Tris Buffered Saline with 0.1% (v/v) Tween 20 1× (TBST) and incubated at RT for 90 minutes. The samples were washed 3× with TSBT 1× for 5 minutes.

The incubation with the primary antibody, T7 tag Polyclonal antibody (Invitrogen) (1:1000 in 1% BSA) was added to slides and incubated at 4 °C O/N, in a dark and humid environment. After rinsed 3× with TBST 1×, the Goat anti-Rabbit IgG (H + L) Cross-Adsorbed Secondary Antibody conjugated with Alexa Fluor® 488 (Invitrogen) (1:40 in 0.2% BSA) was added and it was allowed to incubate in the dark for 90 minutes at RT.

The samples were rinsed with TBST 1× and mounted with one drop of Vectashield® mounting media (Vector Laboratories). The tissue slides were covered with coverslips and observed by fluorescence microscopy using a BX51 microscope (Olympus Portugal SA, Porto, Portugal) coupled with a DP71 digital camera and two sets of filters: FITC – 470–490/520 and TRITC – 530–550/590 (Olympus). All images were acquired using the Olympus cellSens software. Phages were examined in 100-fold enlarged images and identified in larvae in 10-fold enlarged reconstructed images.

Control samples were included. For the positive control, a drop of T7 phage suspension (1 × 10^8^ PFU.mL^−1^) was placed on a microscope slide and incubated at 60 °C for 15 minutes. After that, the dried phage drop was incubated with 4% (v/v) paraformaldehyde (Thermo Fisher Scientific) for 10 minutes at RT, followed by 50% (v/v) ethanol for 10 minutes and allowed to air dry. It was then subjected to the incubation process with primary and secondary antibodies as described above.

#### Statistical analysis

The statistical analysis of the results was performed using GraphPad Prism 6. Mean and standard deviations (SD) were determined for the independent experiments and the results were presented as mean ± SD. Results were compared using Two-way ANOVA, with Turkey’s multiple comparison statistical test. All tests were performed with a confidence level of 95%. Differences were considered statistically different if p ≤ 0.05 (95% confidence interval).

### Ethical approval

This article does not contain any studies with human participants or animals performed by any of the authors.

## Electronic supplementary material

Bacteriophage biodistribution and infectivity from honeybee to bee larvae using a T7 phage model
