# Supplementary material for: Bacteriophage biodistribution and infectivity from honeybee to bee larvae using a T7 phage model
Source: Sci Rep. 2019 Jan 24;9:620. doi: 10.1038/s41598-018-36432-x (PMC6345884; doi:10.1038/s41598-018-36432-x)
Supplement: Supplementary file 1 — Bacteriophage biodistribution and infectivity from honeybee to bee larvae using a T7 phage model [file 41598_2018_36432_MOESM1_ESM.pdf]

Supplementary Information

Bacteriophage biodistribution and infectivity from honeybee to bee larvae using a T7 phage model

Henrique G. Ribeiro[1], Rossana Correia[2,3], Tiago Moreira[4], Diana Vilas Boas[1], Joana Azeredo[1], Ana Oliveira[1]*

[1]CEB - Centre of Biological Engineering, LIBRO - Laboratório de Investigação em Biofilmes Rosário Oliveira, University of Minho, 4710-057 Braga, Portugal.
[2]I3S - Institute for Research and Innovation in Health Sciences, University of Porto, 4200-135 Porto, Portugal
[3]Ipatimup - Institute of Molecular Pathology and Immunology of the University of Porto, 4200-135 Porto, Portugal
[4]BeePrado Unipessoal, Lda., Rua 1, no 32, Ramalha 4730-475 Vila de Prado, Portugal

* Corresponding Author: anaoliveira@deb.uminho.pt

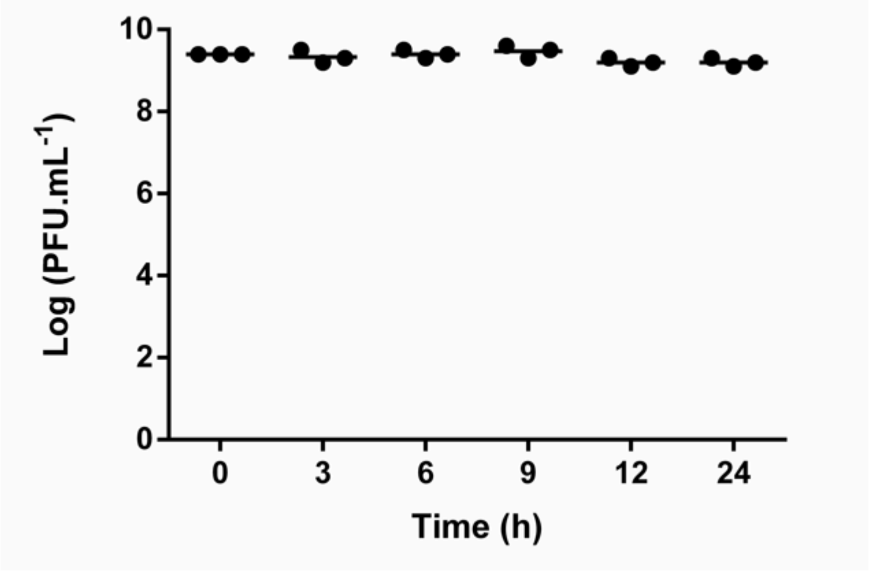

Supplementary Figure S1. Effect of 50% (w/v) sucrose on T7 phage concentration (PFU mL^{-1}). Data shows each of the three independent assays (dark circle) and the average (line). LOD (Limit of Detection) = 2 Log.

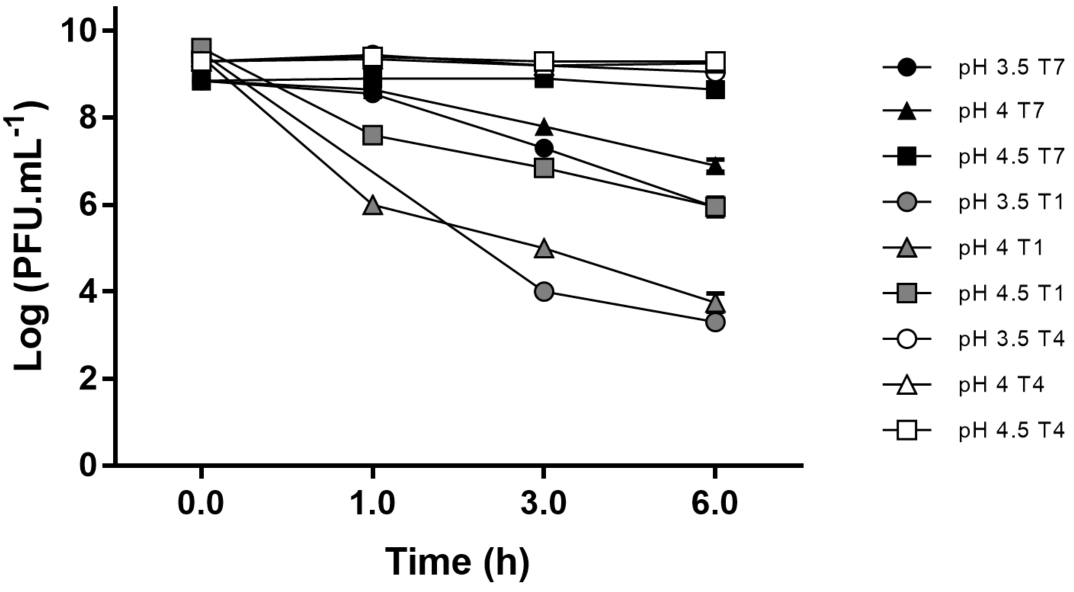

Supplementary Figure S2. Effect of pH in T7 (dark figures), T1 (grey figures) and T4 (white figure) phage concentration (PFU mL^{-1}). Data shows each of the three independent assays performed at pH 3.5 (circle), pH 4.0 (triangle) and pH 4.5 (square). LOD = 2 Log; statistical significance, $p<0.05$.

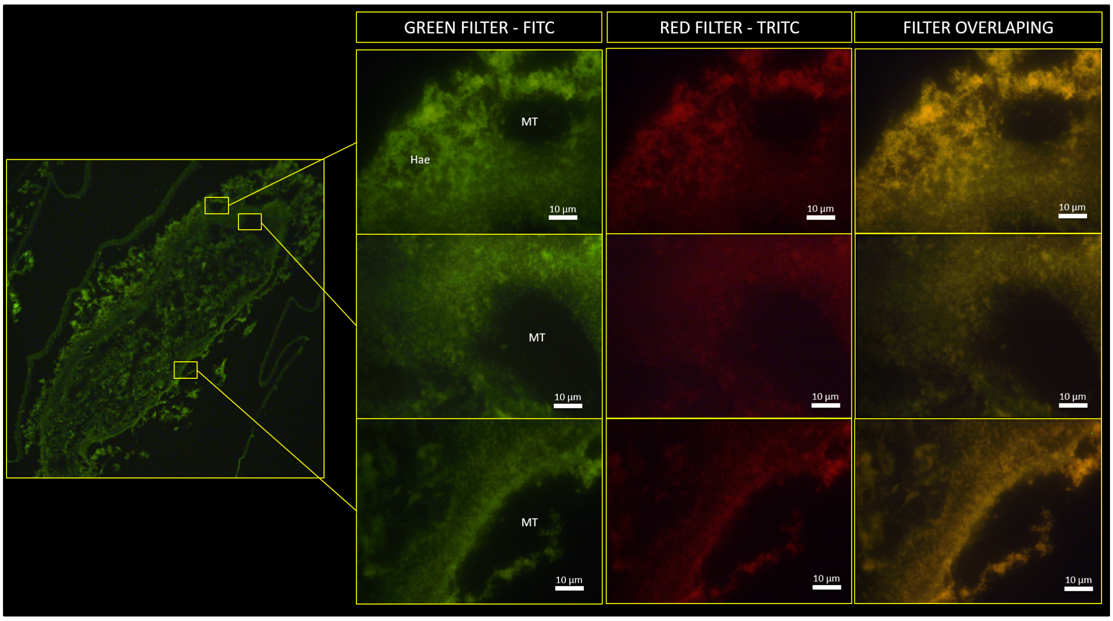

Supplementary Figure S3. Epifluorescence images showing larvae tissue without phage treatment (negative control). Immunofluorescence staining with T7 tag polyclonal antibody and Goat anti-Rabbit IgG (H+L) Cross-Adsorbed Secondary Antibody conjugated with Alexa Fluor® 488 (larvae global image and details with

100x magnitude). The left column show the tissue with no green pixels (FITC filter); the central column show the tissue autofluorescence of tissue (TRITC filter) and the right images present the overlap of the two channels.

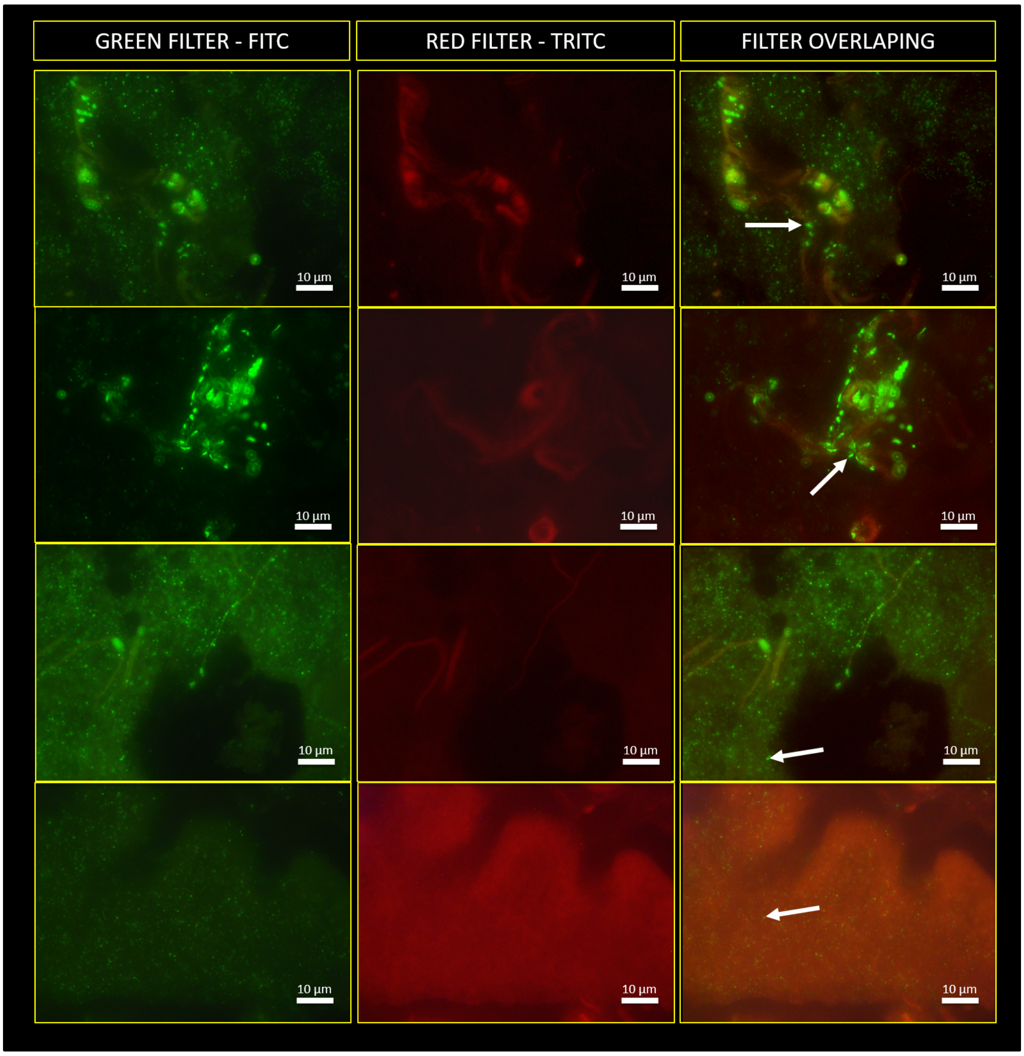

Supplementary Figure S4. Epifluorescence images showing efficient discrimination between the T7 phage-specific bright-green fluorescent signal of Alexa Fluor® 488 (positive control). Immunofluorescence staining with T7 tag polyclonal antibody and Goat anti-Rabbit IgG (H+L) Cross-Adsorbed Secondary Antibody conjugated with Alexa Fluor® 488. The left column show the phage staining (FITC filter); the central column show the tissue autofluorescence (TRITC filter) and the right images present the overlap of the two channels discriminating the T7 phage-specific bright-green fluorescent signal of Alexa Fluor® 488 from the tissue autofluorescence. Arrows indicate phages stained that can easily be visualized on the overlap channel.
